# Prevention of Early Sudden Cardiac Death after Myocardial Infarction Using the Wearable Cardioverter Defibrillator—Results from a Real-World Cohort

**DOI:** 10.3390/jcm12155029

**Published:** 2023-07-31

**Authors:** Ursula Rohrer, Martin Manninger, Lukas Fiedler, Clemens Steinwender, Ronald K. Binder, Markus Stühlinger, Birgit Zirngast, David Zweiker, Andreas Zirlik, Daniel Scherr

**Affiliations:** 1 Division of Cardiology, Department of Medicine, Medical University of Graz, 8036 Graz, Austriadaniel.scherr@medunigraz.at (D.S.); 2 Division of Internal Medicine, Cardiology and Nephrology, Department of Medicine, Hospital Wiener Neustadt, 2700 Wiener Neustadt, Austria; 3 Division of Cardiology, Department of Medicine, University Hospital Salzburg, 5020 Salzburg, Austria; 4 Division of Cardiology and Intensive Care, Department of Medicine, Kepler University Hospital Linz, 4020 Linz, Austria; 5 Division of Cardiology and Intensive Care, Department of Medicine, Hospital Klinikum Wels-Grieskirchen, 4710 Grieskirchen, Austria; 6 Division of Cardiology and Angiology, Department of Medicine, University Hospital Innsbruck, 6020 Innsbruck, Austria; 7 Division of Cardiac Surgery, Medical University of Graz, 8036 Graz, Austria

**Keywords:** sudden cardiac death, implantable cardioverter defibrillator, wearable cardioverter defibrillator, acute myocardial infarction, ventricular fibrillation, ventricular tachycardia

## Abstract

Background: After acute myocardial infarction (AMI), patients are at risk of sudden cardiac death. The VEST trial failed to show a reduction in arrhythmic mortality in AMI patients with an LVEF ≤ 35% prescribed with a WCD, having a lower-than-expected WCD wearing compliance. Objectives: The aim was to investigate on outcomes of patients in a real-world Austrian cohort with good compliance. Methods: A retrospective analysis of all eligible Austrian WCD patients according to the VEST trial inclusion and exclusion criteria between 2010 and 2020 was performed. Results: In total, 105 Austrian patients (64 ± 11 years, 12% female; LVEF 28 ± 6%) received a WCD for a median of 69 (1; 277) days after AMI (wearing duration 23.5 (0; 24) hours/day). Within the first 90 days, 4/105 (3.8%) patients received 9 appropriate shocks (2 (1; 5) shocks). No inappropriate shocks were delivered, and 3/105 (2.9%) patients died during follow-up. Arrhythmic mortality (1.9% Austria vs. 1.6% VEST, *p* = 0.52), as well as all-cause mortality (2.9% vs. 3.1%, *p* = 0.42) was comparable in both cohorts. Conclusions: The WCD is a safe treatment option in a highly selected cohort of patients with LVEF ≤ 35% after AMI. However, despite excellent WCD wearing duration in our cohort, the arrhythmic mortality rate was not significantly different.

## 1. Introduction

Patients are at a significant risk of ventricular arrhythmia (VA) and arrhythmic death in the early phase after acute myocardial infarction (AMI), especially if left ventricular function is significantly impaired. Left ventricular ejection fraction (LVEF) shows an indirect proportional relationship to sudden cardiac death (SCD) and mortality rates [1]; therefore, it is used for risk stratification and as decision support for primary prevention implantable cardioverter defibrillator (ICD) implantation in the current guidelines [2].

Several prospective randomized controlled trials showed that ICD implantation in the early phase after AMI with reduced LVEF may prevent arrhythmic death but simultaneously does not impact all-cause mortality [2,3,4]. The current European Society of Cardiology (ESC) guidelines recommend reevaluation of the left ventricular function 6–12 weeks after AMI when left ventricular dysfunction (LVEF ≤ 40%) is present at discharge. This serves to await the potential recovery of the LVEF and to postpone the decision as to whether ICD implantation is necessary [2,3,4]. Since these patients are unprotected from VA during this period, the wearable cardioverter (WCD) could fill the gap and protect patients from arrhythmic death without the hazards of ICD implantation in the early phase after an AMI [2].

The VEST—Vest Prevention of Early Sudden Death—trial [5] included 2302 AMI patients with a LVEF ≤ 35%, which were randomized in a 2:1 ratio into optimal medical therapy (OMT) alone or OMT combined with a WCD. Although 1.9% of patients received WCD therapies, the trial failed to show a significant reduction in arrhythmic mortality (1.6% with WCD vs. 2.4% with OMT, *p* = 0.52) with WCD therapy in AMI patients. One of the most important reasons may have been the lower-than-expected WCD wearing compliance (median 18 h/day) [5,6]. Analyses suggest that a higher WCD wearing compliance might have positively influenced the outcomes [7].

In this study, we aimed to investigate the incidence of WCD treatments and outcomes of all patients prescribed a WCD with AMI and LVEF ≤ 35% in a real-world national cohort in Austria with good wearing compliance [8].

## 2. Materials and Methods

### 2.1. Austrian WCD Registry

The Austrian WCD registry enrolled 896 patients in 56 centers who received a WCD in 2010–2020. All patients were informed about the nationwide WCD registry at the timepoint of WCD prescription and were asked for informed consent by their treating physician. The study, led by the Medical University of Graz, received institutional review board approval from the local ethics committee.

Baseline characteristics and follow-up data were recorded from medical records; compliance, wearing duration, programming for ventricular tachycardia (VT) and ventricular fibrillation (VF), as well as all alarms and WCD treatments were collected from the ZOLL Patient Management Network and then correlated with medical records via electronic hospital systems. The ZOLL Patient Management Network is a website maintained by the manufacturer of the WCD (ZOLL, Pittsburgh, PA, USA).

### 2.2. Austrian “VEST” Cohort

Within the Austrian WCD registry, we performed a retrospective analysis of all patients that were eligible according to the inclusion and exclusion criteria of the original VEST trial (Table 1) between 2010 and 2020 [5].

### 2.3. WCD Diagnostics and Treatments

Electrocardiogram (ECG) recordings during different alarms either triggered by an automatically working algorithm with predefined detection limits or those manually recorded by the patient were reviewed in the online network.

### 2.4. Automatically Triggered Alarms

The physicians can decide the heart rate limits for VT and VF detection; the default programming for the detection threshold is 150 bpm for VT and 200 bpm for VF. These automatically detected ECGs trigger an alarm so the patient can react to avoid an inappropriate WCD shocks of stable VA or due to false alarms. When VAs are captured but the WCD treatment is aborted by the patient, these arrhythmias are counted as hemodynamically stable VAs unless the correlated medical records disagree.

### 2.5. Manually Recorded Alarms

These alarms are captured by patients pressing the buttons on their WCD when they feel symptoms that may be arrhythmia associated but may not trigger WCD treatments.

#### Appropriate and Inappropriate Treatments

The appropriateness of applied WCD shocks was assessed by three independent electrophysiologists independently assessing the blinded ECG stripes.

Sustained VTs (>30 s), VF, and torsade de pointes were adjudicated as appropriate alarms. Asystole, bradycardia, artefacts, pacemaker oversensing, sinus tachycardia, atrial fibrillation (AF) with rapid ventricular response, or non-sustained ventricular tachycardia (nsVT, <30 s) triggered alarms were categorized as inappropriate.

### 2.6. Follow-Up

All available medical reports and additional information from telephone follow-ups by treating physicians were reviewed. Data concerning arrhythmic events, such as WCD treatments, VA, SCD, or cardiovascular death/death from any cause, were documented in the registry as well as at clinical follow-ups after WCD termination.

The cause of death was categorized as arrhythmic death or death from any cause following medical reports. When the WCD was worn during the event, the ECG recording was included in the adjudication [8]. WCD treatments and mortality were captured until day 90 to be comparable to the outcomes from the VEST trial that had a follow-up period of 3 months defined by the study protocol. Outcomes concerning ICD implantation and other clinical outcomes of the Austrian cohort were captured at the timepoint of WCD termination, which was at day 1–277, with 31/105 patients wearing the WCD longer than 90 days (92–277). This led to a longer observation period in one third of the Austrian patients compared to the VEST trial to document the follow-ups as detailed as possible in this real-life cohort. The adjudication of the outcome events and mortality was assessed by two independent cardiologists.

### 2.7. Data Analysis

Continuous variables are presented as mean ± standard deviation and/or interquartile range, or median and range (min; max). Categorical variables are presented as percentages (%) and counts. The Kolmogorov–Smirnov test was used to test for the normal distribution. Continuous variables were compared with the Student’s *t* test or the Wilcoxon Rank Sum test, frequencies with Chi-square analysis or Fishers’ exact test, and for comparing two or more samples, the Kruskal–Wallis test, as appropriate. Relationships were assessed via correlation and linear regression, and tested with the one-way ANOVA. Two-tailed *p*-values < 0.05 were considered to indicate statistical significance. No adjustments in the *p*-value were performed as this is considered an explorative analysis of a small cohort. Statistical analyses were performed using SPSS 27 (IBM, Armonk, New York, NY, USA).

## 3. Results

### 3.1. Patient Characteristics

The Austrian WCD cohort patients and patients randomized to a WCD in the VEST trial showed similar baseline characteristics concerning age and LVEF (Table 2), but there was a significantly lower percentage of female patients in the Austria WCD cohort (12% in Austria vs. 27% in VEST; *p* = 0.001). In terms of comorbidities, the Austrian cohort showed a higher prevalence of arterial hypertension (91% vs. 65%, *p* < 0.05). In addition, 27/105 (25%) Austrian patients had preexistent AF, 14/105 (13%) had a valvular heart disease defined at least as moderate stenosis or regurgitation, 9/105 (9%) had a history of thromboembolism, and 5/105 (5%) had a history of stroke, with no correlating data from the VEST cohort.

The cohort of patients undergoing revascularization with percutaneous coronary intervention (PCI) was higher in the Austrian cohort (98% vs. 84%, *p* < 0.05), while no Austrian patient was treated with thrombolytics compared to 8% of patients in the VEST cohort (*p* < 0.05). One Austrian patient (1%) and 117 (8%) VEST patients did not undergo any revascularization strategy [5].

### 3.2. WCD Prescription and Compliance

Austrian WCD patients were prescribed with a WCD at a median of seven days (0; 21) after infarction. The median WCD prescription duration was 69 (1; 277) days, with one patient already receiving several shocks on the first day of WCD prescription (day five post-AMI) and were subsequently being monitored in the intensive care unit (ICU), which led to the prescription duration of one day. The longest wearing duration resulted from a patient rejecting an operative revascularization and staying in a prolonged follow-up for ICD evaluation with guideline-directed medical therapy.

Compared to the Austrian WCD registry, in the VEST trial, a prescription of three months was scheduled, but 30% of participants stopped wearing the WCD within one month of randomization. Furthermore, 43% stopped wearing the WCD within two months, and altogether, 80% stopped wearing the WCD earlier than the intended 90 day follow-up period, with 34% of patients not having worn the WCD at any time and 13% having a daily wearing compliance of less than 22 h/day [5,6].

**Figure 1:**
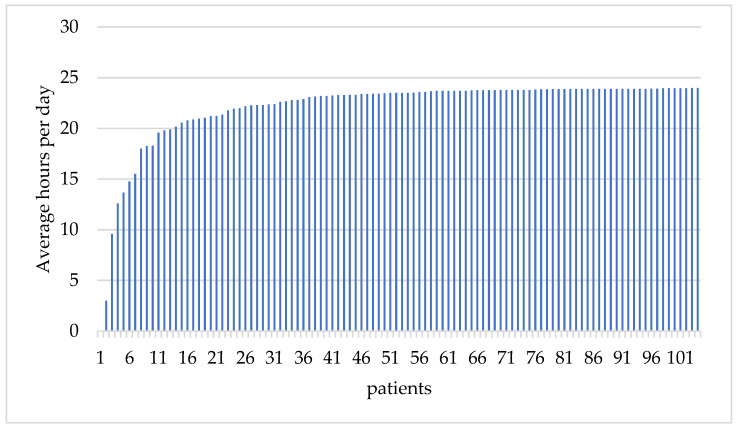
The average WCD wearing duration of the Austrian cohort was 23.5 (0; 24) h/day over 69 (1; 277) wearing days (*n* = 105; WCD = wearable cardioverter defibrillator).

The median wearing duration of the Austrian cohort was 23.5 (0; 24) hours/day, compared to 18.0 (3.8–22.7) hours/day in the VEST trial. Furthermore, 83/105 (80%) had an average wearing duration of 22 h compared to only 53% of patients in the VEST device cohort; 15 patients (14%) had a wearing compliance between 22 and 18 h/day, whereas only 7 patients (6.7%) had a wearing duration below 18 h/day (Figure 1).

### 3.3. WCD Treatments

In total, 4/105 (3.8%) Austrian patients received nine shocks compared to 20/1524 (1.3%) VEST patients who received at least one WCD treatment (*p* = 0.64). The shocks in the Austrian cohort were caused by seven VT and two VF events. The time from event onset to shock was 35 (29; 110) seconds. All events in Austrian patients were terminated to sinus rhythm with the first shock. Of the patients who received WCD treatment, the per patient shock rate in the Austrian cohort was 2 (1; 5). Furthermore, 100% of all shocks (9/9) were delivered within the first 2 weeks after prescription, with a median time from prescription to shock being 7 (1; 12) days. All patients were admitted to the local ICU and stabilized if needed. Only one of them received amiodaron as antiarrhythmic therapy; the other patients did not receive additional antiarrhythmic therapy. All patients were under maximally tolerated betablocker treatment as part of their heart failure treatment. Consequently, all three of these patients were implanted with an ICD as soon as possible depending on surgical capacities and in a average of 15 ± 3.9 days after WCD prescription. One patient died one day after an effective WCD shock in an electrical storm in the ICU.

### 3.4. Total Mortality

In total, 3/105 (death from any cause: 2.9%) patients died during the regular WCD follow-up of 90 days wearing their WCD. Two patients died in an electrical storm, adjudicated as arrhythmic death. These patients wore the WCD that appropriately detected and treated VT/VF, when not aborted by patient. At the timepoint of death due to the electric storm, both patients did not wear the WCD while being monitored and treated in the local ICU. One patient died due to asystole adjudicated as non-arrhythmic death, while no autopsy took place to identify the exact cause of death.

### 3.5. Non-Arrhythmic Mortality

One patient had an out-of-hospital cardiac arrest at the age of 57 while wearing the WCD. He died with asystole as the primary rhythm despite intense resuscitation attempts. There was no prior electrophysiological hint for bradyarrhythmia. This patient had an AMI with 3-vessel-coronary artery disease, an initial LVEF of 35%, and had been fully revascularized.

### 3.6. Arrhythmic Mortality

In total, 2/105 patients (arrhythmic mortality, 1.9%) died due to a ventricular storm. One patient had a large ventricular aneurysm, a severely reduced LVEF of 15%, and thrombi in both ventricles. He had a single-vessel disease and he experienced recurrent monomorphic VTs after being revascularized. After WCD prescription, he aborted three WCD treatments again for monomorphic VTs (300 ms tachycardia cycle length) before being admitted at the ICU. The treating physicians decided to transfer him to a university hospital for VT ablation and evaluation for a left ventricular assist device (LVAD) and/or heart transplantation (HTX). Ventricular thrombi resulted in endocardial ablation and LVAD implantation becoming impossible as cardiac surgeons could not remove the aneurysm due to its large size. He was not eligible for HTX because of ongoing drug abuse. Within a short time span, the situation deteriorated quickly, and the patient died in a refractory electrical storm at the age of 34 years.

The other patient was primarily experiencing stable nsVTs and was being monitored at the cardiology ward until he experienced VF with a WCD shock whilst still being hospitalized more than 48 h after AMI and revascularization. The VF was induced by short-coupled r-on-t premature ventricular contractions. The day after the WCD treatment, the patient died at the age of 64 in an electrical storm and was not responsive to extensive resuscitation attempts at the ICU unit. This patient had a non-ST-elevation AMI (non-STEMI) with two-vessel disease and was fully revascularized with an initial LVEF of 25%.

The WCD was removed at the timepoint of hospitalization during the actual event in both cases due to monitoring at the ICU. The clinical details were retrieved from medical records. All patients with a fatal outcome had VA before WCD prescription.

The total mortality of the VEST device cohort was 3.1% (48/1524); 1.4% (21/1524) were adjudicated as non-arrhythmic and 25/1524 (1.6%) died an arrhythmic death, and only 9 of these 25 patients died while wearing their WCD, capturing a VA. The cause of 2/1524 (0.1%) deaths were not sufficiently documented; therefore, they were adjudicated as “indeterminate” [5,6].

In the Austrian cohort of patients, no inappropriate shock (0/105, 0%) was detected, while 9/1524 patients (0.6%) received inappropriate WCD treatments in the VEST device cohort (*p* = 1.0).

### 3.7. Follow-Up

**Figure 2:**
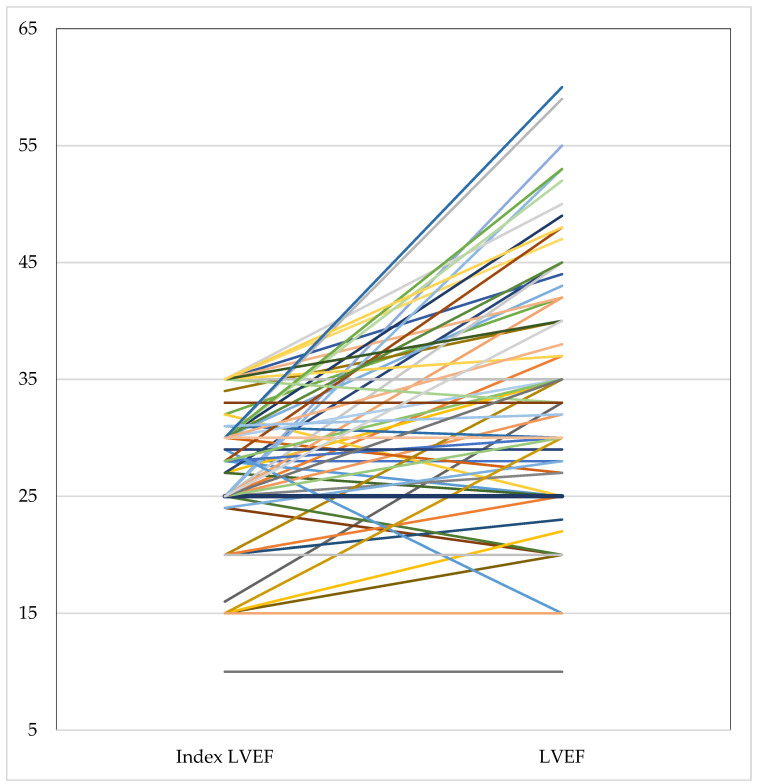
Change in LVEF at the timepoint of prescription (index LVEF) and LVEF after the WCD wearing period. Every line with a different colour stands for the LVEF of an individual patient. (LVEF = left ventricular ejection fraction, WCD = wearable cardioverter defibrillator).

The LVEF at WCD termination was 35% (15; 59) compared to 28% (10; 35) at baseline (Figure 2). An ICD implantation after WCD termination was performed in 46/105 Austrian patients (44%), and of these, 42/105 (40%) had an indication for primary prevention, and 4/105 (4%) for secondary prevention. Furthermore, 38/105 patients (36%) did not receive an ICD at the end of WCD prescription.

In total, 67/1524 (4.4%) VEST patients received ICD implantation in the VEST cohort during follow-up: 19/1524 (1.2%) received an ICD for secondary prevention, 5/1524 (0.3%) were implanted with a cardiac resynchronization therapy device with a defibrillator, 24/1524 (1.6%) were implanted with an ICD for primary prevention, which implicated a protocol deviation within the first 90 days after AMI in the VEST trial, and the reason for ICD implantation was not documented in 19/1524 (1.2%) patients. ICD implantation after the predefined 90 days of follow-up was not documented in the VEST trial.

### 3.8. Alarms

In the Austrian WCD registry, 617 manual events were triggered by 65/105 patients (61%). The recorded ECGs showed normal sinus rhythm in 614/617 ECGs (99.5%), no sustained VA were detected.

**Figure 3:**
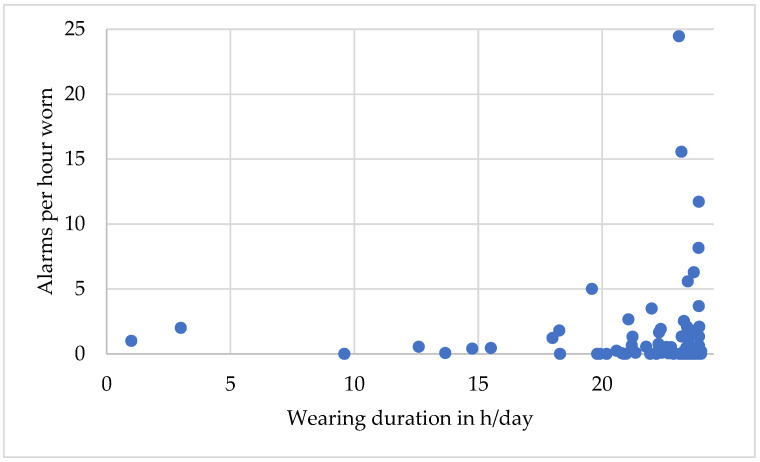
Average number of alarms per hour of the individual patients compared to the wearing duration in hours per day (23.5 h (0; 24 h)).

In addition, 2788 automatically recorded alarms in 76/105 (72%) Austrian patients were triggered by the WCD compared to 57,451 alarms occurring in 72% of VEST device patients. Furthermore, 97.5% (2721/2788) of all automatically recorded alarms were adjudicated as inappropriate. There was no significant correlation between the average number of alarms per hour and compliance, as measured by the average daily wearing time (Spearman’s rho = −0.11, *p* = 0.28) In contrast, patients being confronted with the highest number of alarms indeed showed good compliance (Figure 3).

The proportion of hemodynamically stable VTs without shocks (5/105 (5%) Austrian patients compared to 69/1254 (5.5%) in the VEST cohort) and asystole events (1/105 (1%) Austrian patient compared to 6/1524 (0.4%) VEST patients) within these alarms were comparable in both groups.

## 4. Discussion

The VEST trial did not show a significant reduction in arrhythmic mortality in WCD patients after AMI, with a lower-than-expected compliance as one possible explanation. We compared these results to data from a well-compliant real-world population and demonstrated that shock rates and arrhythmic mortality were similar to those in the VEST trial, despite good WCD compliance.

The primary outcome in the VEST trial was to investigate arrhythmic mortality, which was not different in the Austrian WCD registry (1.9% vs. 1.6%, *p* = 0.52), with a similar all-cause mortality (2.9% in Austria vs. 3.1%, *p* = 0.42).

Earlier trials with similar ICD cohorts already showed that appropriate shocks do not decrease but rather increase mortality rates with a linear association between mortality and shock rates [9,10,11]. Even if the differences in shock rates and mortality rates are not significantly different, which may be due to a lower patient number in the Austrian registry, the following aspects should be considered.

Only 12 patients (23% of all deaths) in the WCD arm of the VEST trial did wear the WCD at the time of their death, and in only 6/12 patients, VT or VF (6/1524, 0.4%) were detected and appropriately treated by the WCD. In addition, a further trial showed that the education of the patient and their wearing compliance are essential for the appropriate detection and treatment of malignant VA [12].

Several trials and registries have already shown the effectiveness of treating malignant VA with a WCD shock [8,12,13,14,15], while the protection against arrhythmic death while not wearing the WCD is obviously not given, as well as the possibility to adjudicate “arrhythmic death” via ECG tracings.

In the VEST trial, all arrhythmic deaths in the VEST trial were adjudicated by an independent panel of experts without reviewing the ECGs recorded by the WCD. The Austrian WCD registry relies on WCD captured ECG tracings correlated with medical records, and exclusively relies only medical records alone when ECG tracings from the WCD were missing (see Supplementary Appendix of the VEST trial [5,8]). In several patients, the emergency medical system detected non-shockable rhythms in the device group of VEST patients not wearing the WCD that needed resuscitation, which might result from the delay between the emergency medical call and timepoint of the first recording of a defibrillator/ECG [5,6].

The VEST investigators also performed an as-treated and per-protocol analysis of their data and mentioned a statistically significant reduction in arrhythmic mortality in the small group of well-compliant patients (525/1524), which incorporated several factors without publishing the specific numbers of WCD treatments and mortality rate; this renders a comparison with our registry data impossible [6]. Nevertheless, arrhythmic deaths occurred in 9/525 patients wearing a WCD with 4 patients not having malignant VA on the WCD at the timepoint of death, as mentioned in the appendix of the VEST trial [5]. Thus, only 5/1524 patients had an arrhythmic event documented and treated by the WCD and died consecutively from what would be the definition of an arrhythmic death in the Austrian WCD registry. In summary, 5/525 compared to 1/105 in the well-compliant cohorts of both trials did not show a significant difference.

We compared the well-compliant Austrian WCD cohort with the original VEST device group with a significantly lower adherence. Previous studies showed that patients in real-world registries have a higher WCD compliance compared to patients in the VEST trial, which might be interlinked with the randomization process within the VEST trial [15,16,17,18]. Previous studies showed that the compliance of the Austrian cohort is consistently high following thorough nurse-based patient education [8]. Thorough patient education and regular follow-ups increase WCD wearing compliance and improve prognosis, especially in the post-AMI population where adherence to medication is of great importance [19].

Comparing the baseline characteristics, there is a low percentage of female patients in both trials. Our real-life registry data shows only 12% of females in the Austrian cohort, which is consistent with findings in large trials investigating on SCD, ICDs, or AMI, where the number of female patients is typically much lower compared to male patients [20,21].

The incidence of coronary artery disease and AMI is significantly lower in female patients in the general population, and ischemic heart disease develops later compared to male patients. Women after the age of 75 are more likely to experience AMI compared to men. There are several assumptions that important risk factors, such as arterial hypertension and hyperlipidemia, are less frequently present in premenopausal woman. Furthermore, the pathophysiology of AMI is different; microvascular changes, distal embolization after plaque erosion, coronary spasms, and spontaneous coronary artery dissection are more frequent compared to plaque rupture with thrombus formation of an epicardial coronary artery in male patients [22].

Apart from a lower incidence, female patients might present with atypical symptoms and are less likely to receive revascularization, while the ESC states that patients benefit from therapy irrespective of their gender [23]. That also might account for a lower number of patients, as patients might not be diagnosed with AMI but rater with ischemic heart disease and were not included in this analysis.

Moreover, as women have a higher incidence of AMI when reaching the age of 75, this age group will be less likely to be considered for a device due to their life expectancy and scarce recommendations on devices in the octogenarians.

Investigations on gender differences in WCD patients reconfirmed that female patients are persistently underrepresented in trials concerning SCD, and they seem to be considered less often for cardiac devices and cardiac procedures in real life [24].

High numbers of inappropriate alarms due to artefacts, sinus tachycardia, AF with a high ventricular rate, or supraventricular tachycardia still pose a problem in patients with WCD as the detection algorithms depends on the surface ECG captured by the WCD. Even though 72% of patients are confronted with alarms, this does not negatively impact WCD wearing compliance. In contrast, patients with a high number of alarms tended to have very good compliance in the Austrian registry. One explanation may be that these patients seek medical help more often and are more compliant concerning medication intake due to a higher awareness of their disease. As visits to any medical institution out of hospital were not captured, this hypothesis still needs to be confirmed.

Within all reviewed alarms, the rate of WCD initiated appropriate shocks for malignant VA was higher in the Austrian than in the VEST cohort (3.8% vs. 1.3%), while the difference did not reach statistical significance (*p* = 0.64).

A previous meta-analysis confirmed a higher event rate in observational studies compared to the VEST trial [25]. These findings support the hypothesis that more VA are captured and more WCD treatments are applied when the device is worn continuously.

Reviews analyzing the studies conducted so far and data from registries suggest that a WCD is an effective treatment option to bridge the time until an ICD can be implanted [26]. To apply this recommendation to the VEST cohort, patients would either need a reduced LVEF ≤ 35% without potential to recover or experience VA > 48 h after AMI. Patients with these characteristics might benefit from a WCD due to the even higher SCD, but should still be studied in a randomized trial.

Furthermore, randomized studies including well-compliant patients after AMI with additional risk factors could identify a cohort benefiting from a WCD after AMI and gather more specific indications for prescription. Such risk factors could be, for example, reduced LVEF during follow-up or present before AMI, the patient being not fully revascularized, nsVTs during the in-patient monitoring phase, as well as imaging data to identify a potential substrate for VA, which could be incorporated to identify patients with a very high SCD risk. Additionally, factors that predict low WCD wear time should also be considered as the effectiveness is dependent on the wear time. Factors such as LVEF ≤ 25%, prior diagnosis of heart failure or diabetes, and a WCD shock within the first week of prescription irrespective of whether appropriate or inappropriate, have been identified in the post-hoc analysis of the VEST trial [6,27]. As the rate of adverse events and inappropriate shocks is low, WCDs might already be supplied in cohorts with a specifically high risk, even without evidence from large trials, but most patients might not benefit from a WCD after AMI concerning arrhythmic mortality.

A review discussing the results of the VEST trial suggests that another function of the device may add a benefit for mortality, the monitoring function. The WCD was initially designed to prevent SCD but does also record nsVTs and non-malignant arrhythmia or bradycardia that might need further treatment. Early treatment of arrhythmias, such as tachycardic conducted AF, can be crucial in patients with HFrEF. Moreover, the recorded ECGs and information may help in further risk stratification and raise awareness to the patient concerning their underlying disease. As a conclusion, the authors recommend further prospective studies, which is consistent with results from a previous study [27].

Another factor that needs to be considered is the updated heart failure guidelines that recommend early initiation of angiotensin receptor neprilysin inhibitors and sodium-glucose cotransporter-2 inhibitors. The new recommendations from the 2021 ESC guidelines for the diagnosis and treatment of acute and chronic heart failure might not only influence cardiovascular outcomes [28].

One large burden is still the financial part concerning reimbursement, which is still not solved in all countries. Randomized trials to gather evidence might not be attractive for investigators or sponsors, but positive outcomes would support that reimbursement if specific indications are necessary [19]. The evidence to justify the expanding use in larger cohorts requires randomized controlled prospective trials as the data so far, apart from the VEST trial, is derived from observational studies and registries. This study design is influenced by the diversity of the reported patients and the lack of control groups [29].

## 5. Limitations

This study is subject to several limitations. The study was performed retrospectively, and was non-randomized and non-blinded, which was necessary to assess the everyday benefit of a WCD in patients after AMI with reduced LVEF in the real-world. In a retrospective analysis of 105 patients, there may be statistical limitations concerning power and alpha error inflation due to the relatively small sample size, potentially leading to a reduced ability to detect true effects and an increased risk of false positive results.

The Austrian nurse-based training approach results in good adherence following profound patient education. This data reflects Austrian patients with similar initial WCD training being prescribed in 1 of the 56 included centers, while patient education takes place in a different manner in every health care system.

Women are underrepresented in clinical trials concerning SCD and cardiac implantable electronic devices as well as in real life and need to be evaluated carefully as they are presumably underdiagnosed and undertreated. Female patients have a higher event rate as analyses of female WCD subgroups show even higher event rates, and therapies are equally effective [30].

## 6. Conclusions

Real-world data from the Austrian WCD registry showed the high effectiveness of WCD treatments but could not support an impact on arrhythmic mortality in a well-trained and compliant WCD cohort, as opposed to the VEST study cohort.

## Figures and Tables

**Table 1:** Inclusion and exclusion criteria of the VEST ** trial. (1) This table shows the inclusion and exclusion criteria of the VEST trial (* CABG = coronary artery bypass grafting, † ICD = implantable cardioverter defibrillator, ‡ LVEF = left ventricular ejection fraction, § AMI = acute myocardial infarction, ‖ PCI = percutaneous coronary intervention, # STEMI = ST-elevation myocardial infarction, ** VEST = “Vest Prevention of Early Sudden Death” trial, †† WCD = wearable cardioverter defibrillator).

Inclusion Criteria	Exclusion Criteria
Patients identified in the hospital or within 7 days after discharge with a diagnosis of an acute MI § (STEMI # or non-STEMI #) LVEF ‡ ≤ 35%, determined at the following timepoints: - if no PCI ‖, ≥8 h after AMI § - if acute PCI ‖ occurs, ≥8 h after PCI ‖ - if CABG * is planned (before or within 7 days of discharge), most recent assessment at least 48 h post-CABG *. Age ≥18 years	Existing ICD or indication for an ICD † Existing unipolar pacemakers/leads Chronic renal failure requiring hemodialysis after hospital discharge Chest circumference too small/large for WCD †† Participants discharged to a skilled nursing facility with anticipated stay > 7 days Pregnancy Inability to consent Any condition/circumstance that renders the participant unsuitable for the study

**Table 2:** Baseline characteristics. This table shows the baseline characteristics of the Austrian WCD cohort and the VEST device cohort, and the comparison of both. (* CABG = coronary artery bypass grafting, † LVEF = left ventricular ejection fraction, ‡ PCI = percutaneous coronary intervention, § VEST = “Vest Prevention of Early Sudden Death” trial, ‖ WCD = wearable cardioverter defibrillator).

	Austrian WCD ‖ Cohort	VEST § WCD ‖ Cohort [5]	*p*-Value
Number of patients	105	1524	-
Age	64 ± 11 years	61 ± 13 years	n.s.
Female	13/105 (12%)	413/1521 (27%)	<0.01
Arterial hypertension	96/105 (91%)	994/1521 (65%)	<0.001
Diabetes mellitus	27/105 (25%)	497/1521 (32.7%)	n.s.
PCI ‡	103/105 (98%)	1275/1513 (84.3%)	<0.001
CABG *	1/105 (1%)	14/1513 (0.9%)	n.s.
Thrombolytics	0/105 (0%)	118/1513 (7.8%)	<0.01
LVEF † baseline	28 ± 6%	28.2 ± 6.1%	n.s.

## Data Availability

Data available on request due to restrictions, e.g., privacy or ethical.
